# Torquetenovirus detection in exosomes enriched vesicles circulating in human plasma samples

**DOI:** 10.1186/s12985-018-1055-y

**Published:** 2018-09-20

**Authors:** Francesco Martelli, Lisa Macera, Pietro Giorgio Spezia, Chiara Medici, Mauro Pistello, Daniele Guasti, Paolo Romagnoli, Fabrizio Maggi, Simone Giannecchini

**Affiliations:** 1 Department of Experimental and Clinical Medicine, University of Florence, Viale Morgagni 48, I-50134 Florence, Italy; 2 Virology Unit, Pisa University Hospital, Pisa, Italy; 3 Retrovirus Center and Virology Section, Department of Translational Research, University of Pisa, Pisa, Italy

**Keywords:** Anelloviruses, Torquetenovirus, Exosomes, DNA viral load, Viral persistence, HIV, transplant recipients

## Abstract

**Background:**

Torquetenovirus (TTV) belongs to *Anelloviridae* family, infects nearly all people indefinitely without causing overt disease establishing a fine and successful interaction with the host. Increasing evidence have shown some human viruses exploit extracellular vesicles thereby helping viral persistence in the host. Here, the presence of TTV in extracellular vesicles circulating in human plasma was investigated.

**Methods:**

TTV DNA was quantified in plasma-derived exosomes from 122 samples collected from 97 diseased patients and 25 healthy donors. Exosomes enriched vesicles (EEVs) were extracted from plasma and characterized by Nanoparticle tracking analysis, by western blot for presence of tetraspanin CD63, CD81 and annexin II protein and, finally, by electron microscopy (EM). Presence and quantitation of TTV DNA were assessed with an universal single step real-time TaqMan PCR assay.

**Results:**

Preliminary investigation showed that the human plasma extracted extracellular vesicles exhibited a main size of 70 nm, had concentration of 2.5 × 10^9^/ml, and scored positive for tetraspanin CD63, CD81 and annexin II, typical characteristic of the exosomes vesicles. EEVs extracted from pooled plasma with TTV DNA viremia of 9.7 × 10^4^ copies/ml showed to contain 6.3 × 10^2^ TTV copies/ml, corresponding to 0.65% of total viral load. Important, TTV yield changed significantly following freezing/thawing, detergents and DNAse treatment of plasma before EEVs extraction. EEVs purified by sucrose-density gradient centrifugation and analysis of gradient fraction positive for exosomes marker CD63 harbored 10^2^ TTV copies/ml. Moreover, EM evidenced the presence of TTV-like particles in EEVs. Successive investigation of plasma EEVs from 122 subjects (37 HIV-positive, 20 HCV infected, 20 HBV infected, 20 kidney transplant recipients, and 25 healthy) reported TTV DNA detection in 42 (34%) of the viremic samples (37 were from diseased patients and 5 from healthy people) at a mean level of 4.8 × 10^3^ copies/ml. The examination of EEVs selected samples reported the presence of TTV genogroup 1, 3, 4 and 5, with genogroup 3 highly observed.

**Conclusions:**

Collectively, although these observations should be confirmed by further studies, circulation of TTV particles in EEVs opens new avenues and mechanistic insights on the molecular strategies adopted by anelloviruses to persist in the host.

## Background

Torquetenovirus (TTV), first identified in 1997, is the prototype of a vast array of naked, small viruses with similar genomes that continue to be added to the list of agents causing chronic productive infections and high levels of plasma viremia in humans [1, 2]. All these viruses are presently classified in the newly established *Anelloviridae* family [3]. Many remarkable properties of TTV are now well known including its DNA genome, a particularly small single-stranded circular molecule of about 3.8 kilobases with negative polarity that consists of at least 4 open-reading frames and an untranslated region (UTR) containing several highly conserved sequences showing over 90% identity among TTV isolates [2, 4]. Moreover, TTV UTR has the potential to encode microRNAs (miRNAs), small noncoding 22 nucleotide-long RNAs that are thought to play a role in evading immune response and regulating viral reactivation and pathogenesis [5, 6]. The virus is astonishingly prevalent in humans and exists in numerous genetic species (at least 29 species have been identified so far), each one consisting of a large number of strains. Thus an amazing feature of TTV is that it circulates as a mixture of a varying number of species in the blood and many other tissues of nearly all people for long periods or indefinitely. The overall load of TTV in blood varies widely as a result of how actively the virus replicates in T lymphocytes that are probably the major site of viral replication, although other cell types outside of the hematopoietic compartment may also contribute to the viral burden [7–9]. Imbalance of the immune system has a significant impact on replication of TTV [10–13]. HIV-1-infected individuals and other immunocompromised people present higher prevalence rates and/or higher concentrations in blood of TTV than healthy controls, arguing for the existence of a correlation between severity of the patients’ immunosuppression and burdens of TTV carried. Again, in HIV positive patients, an association between high TTV loads, on one hand, and low CD4 T cell counts, high HIV viral loads, and overt AIDS on the other has been evidenced in several reports [14–17]. Clearly, these together with additional studies [18–20] have corroborated the idea that the poorly functional immune system of HIV-1 infected or other immunocompromised patients permits to the TTV to replicate in the host more freely than would occur if the immune system was functionally intact. Thus, it has been recently proposed of using TTV viremia to gauge global immune function in infected subjects [21]. At present, no human disease has yet been linked with certainly to the direct action of TTV, which is instead the most representative and abundant component of the human virome [22].

Many aspects of the natural history and pathogenesis of this under many respects surprising virus are still poorly understood. The life cycle of TTV in the infected host and how the virus can be scattered so extensively in human body is no exception.

Extracellular vesicles (EVs) are a heterogeneous group of membrane vesicles secreted by almost all cell types and, according to the mechanism of generation, they can be distinguished in multiple classes including exosomes (30–150 nm vesicles produced in the endocytic pathway, accumulated in large multivesicular bodies and delivered from their fusion with the cell membrane), ectosomes (100–1000 nm vesicles formed by the direct budding from the plasma membrane or released by the double-membraned autophagosomes fusion with the plasma membrane), and apoptotic bodies (released upon cell fragmentation during apoptotic cell death) [23, 24]. EVs contribute to cell-to-cell communication and other processes being also potentially implicated in cancer cell signaling, inflammatory conditions, and immune regulation. Functions and cargos of EVs are determined by their different subcellular origin [24–26]. Increasing evidence demonstrates that cells infected by different viruses may secrete EVs containing several viral components but also, in some instances, infectious virus, and that a number of human viruses, such as human immunodeficiency virus (HIV), hepatitis A (HAV), B (HBV), C (HCV), and E (HEV) viruses, herpes simplex virus, and Epstein-Barr virus, use EVs for providing additional route of transmission, escaping from immune recognition, and facilitating their persistence into the infected host [27, 28].

The recent finding that TTV encoded miRNAs are found in plasma EVs at expression levels with wide individual [29] variability suggests the importance of in depth investigation on the possible role of EVs in TTV pathogenesis. Thus, aim of this study was to demonstrate TTV presence in exosomes enriched vesicles (EEVs), to quantify the EEVs associated virus, and to compare TTV loads in EEVs and plasma samples of healthy controls and patients with different pathological conditions.

## Methods

### Samples

A total of 122 randomly selected subjects were studied. Ninety-seven were diseased patients who were referred to our laboratories for routine virological analyses; the remaining 25 included healthy blood donors. The diseased patients were 57 immunosuppressed subjects (37 with HIV infection, and 20 kidney transplant recipients), and 40 subjects affected by liver pathologies (20 with HCV infection, and 20 with HBV infection). Blood samples were obtained by venipuncture, aliquots were immediately prepared, stored and kept under sterile conditions at − 80 °C until use. The study was approved by ethics committee at Pisa University Hospital, Pisa.

### EEVs extraction and characterization

EEVs were isolated from 1 ml of plasma after centrifugation at 14,000 x *g* per 20 min by using an exosome specific extraction kit (Norgen Biotek Corp., Thorold, ON, Canada), following the manufacturer’s protocol [30, 31]. EEVs characterization was performed with the Nanoparticle tracking analysis (NTA) using the Nanosight NS300 system (Malvern Instruments Ltd., Malvern, United Kingdom) equipped with a sCMOS camera and a blue laser (488 nm) to illuminate the particles within the size range of 10–2000 nm. The sample was loaded into the analysis chamber using a syringe pump at a constant flow rate. Nanoparticles were illuminated by the laser and their movement under Brownian motion was tracked for 60 s at camera level 12. Five videos were captured to provide significative concentration and size data. During the analysis, a viscosity of water and a detection threshold at pixel value 7 were set. All videos were subjected to NTA using the Nanosight particle tracking software to provide nanoparticle concentration and size distribution profiles. The software tracks many particles individually and using the Stokes-Einstein equation calculates their hydrodynamic diameters. Additionally, according to the International Society for Extracellular Vesicles statement [32], the amount of different categories of proteins were investigated in EV preparations. The proteins characterized were the tetraspanins CD63 and CD81, the membrane-binding protein annexin II, and the protein cytochrome P450. The analytic approach was carried out by Western blot (WB) analysis of EEVs electrophoresed on 10% SDS-PAGE and then probed with anti-CD63, anti-CD81, anti-annexin II and anti-cytochrome P450 monoclonal antibodies followed by peroxidase-conjugated anti-mouse IgG polyclonal antibody.

### Electron microscopy

EEVs extracted samples were fixed with 2% formaldehyde and 2.5% glutaraldehyde in 0.1 mol/L cacodylate buffer, pH 7.4, osmicated and embedded in epoxy resin. Sections (~ 70 nm thick) were stained with gadoliniom acetate (Electron Microscopy Sciences, Hatfield, PA) and bismuth subnitrate and observed in a JEM 1010 (Jeol, Tokyo, Japan) at 80 kV [33, 34]. Photomicrographs were taken with a digital camera MegaView III (Soft Imaging System, Muenster, Germany) connected with a personal computer (Dell, Round Rock, Texas) with dedicated software (AnalySIS, Soft Imaging System, Muenster, Germany).

### Ultracentrifugation in sucrose density gradient

Plasma sample from pooled HIV/TTV coinfected patients and purified EEVs was layered onto a discontinuous density gradient consisting of 60, 40, 30, 20, 10% (wt/vol) sucrose dissolved in autoclaved and filtrated (0,22 um) sterile water in a polyallomer thinwall tube (Beckman Co, Palo Alto, CA) with a nominal capacity of 14 mL. The tube was overlaid with sterile PBS and centrifuged at 129300 x g for 18 h at 4 °C in a Beckman SW40Ti rotor (Beckman Co, Palo Alto, CA). Each fraction were handle collected from the surface and subsequently were tested for EEVs marker by WB analysis and TTV quantitative real-time PCR.

### TTV quantification and genetic characterization

TTV infection was assessed by extracting viral DNA from 200 μl of plasma samples and 200 μl of EEVs (obtained from 1 ml of same plasma samples) using QIAamp DNA Mini kit (QIAGEN, Chatsworth, CA) and determining presence and load of TTV genome using a single step universal TaqMan real-time PCR assay [35]. As described, PCR target is a highly conserved fragment of the untranslated region (UTR) of the TTV genome and the assay is therefore capable detecting all TTV species hitherto described. The sensitivity of the real-time assay was of 10 copies per ml of plasma.

Selected plasma and EEVs samples found positive by the UTR real-time PCR assay were amplified by five PCR protocols, each specific for one TTV genogroup [35]. PCR assays are targeted on either the UTR (genogroups 4 and 5) or open reading frame (ORF) 1 genes (genogroups 1, 2, and 3) of the viral genome. Sensitivity of each genogroup-specific assay has been previously tested and found to be of about 1000 copies per ml of plasma [35].

### Computer analysis of viral late domain in TTV capsid ORFs gene

TTV capsid ORF translated sequences from all of 29 TTV species recognized were scanned for the presence of late assembly domains by ScanProsite Tool from ExPASy Bioinformatic Resource Portal (https://prosite.expasy.org/scanprosite/).

### Statistical analysis

SPSS software version 23 (IBM, Chicago, IL, USA) was used for statistical analysis. Fisher’s exact test was applied to evaluate the heterogeneity of contingency tables. Differences between distributions were calculated by using non-parametric Mann-Whitney U and Kruskal-Wallis tests. Correlations between continuous non-normally distributed variables were assessed using Spearman rho correlation coefficient. All *p* values presented are based on two-tailed tests, and *p* < 0.05 was considered statistically significant.

## Results

### Studies on TTV DNA presence in plasma EEVs

**Fig. 1:**
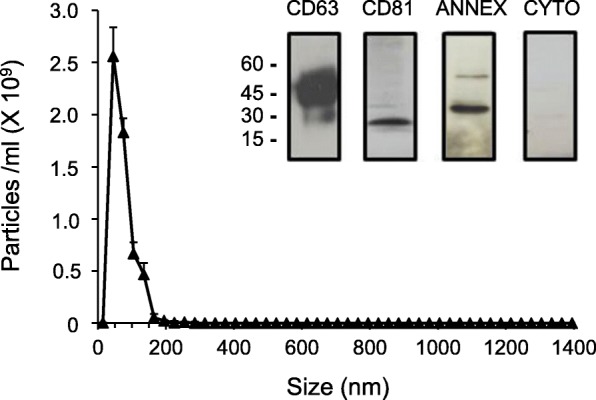
Size, concentration and markers of plasma-derived EEVs. The EEVs purified from pooled plasma samples of HIV were analyzed with the NanoSight NS300 Nanoparticle analysis system. Western blot profiles of CD63, CD81, annexin II and cytochrome P450 from the same EEVs were electrophoresed on 10% SDS-PAGE and then probed with anti-CD63, anti-CD81, anti-annexin II and anti-cytochrome P450 monoclonal antibodies followed by a peroxidase-conjugated anti-mouse IgG polyclonal antibody. The values shown are the means ± standard deviations of 3 independent experiments

**Fig. 2:**
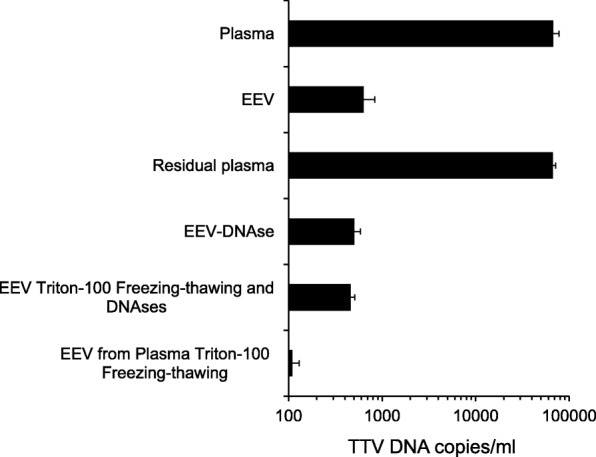
TTV DNA quantification in plasma-derived EEVs. TTV load in whole plasma (Plasma), EEVs (Exosomes enriched vesicles) and residual plasma fraction after EEVs extraction (Residual) are reported. TTV loads in EEVs after DNAse treatment (EEV-DNAse), in EEVs before or after freezing/thawing and detergent treatment, and in EEVs extracted from pooled plasma after freezing/thawing and detergent treatment are also indicated. The values shown are the means ± standard deviations

Despite the knowledge of TTV as a ubiquitous human virus, nothing is known about the possibility that the virus can be spread into the body by EVs. For studying the issue, firstly plasma samples from different HIV/ TTV coinfected patients were pooled and the pooled plasma was used in order to confirm the nature of isolated vesicles. As shown in Fig. 1, extracted vesicles exhibited typical characteristics of exosomes: they had a particle size of about 70 nm in diameter, a total concentration of 2.5 × 10^9^ particles per ml of plasma, and they resulted highly enriched in tetraspanins CD63 and CD81 and in annexin II, but they resulted negative for the cytochrome P450 protein [32]. Then, TTV DNA was detected and quantified by real-time UTR PCR in the well characterized EEVs isolated from pooled plasma. As shown in Fig. 2, the total plasma, assayed before EEVs extraction, contained TTV DNA at the level of 9.7 × 10^4^ copies per ml. Of these DNA copies, a part corresponding to 6.3 × 10^2^ copies per ml (0.65% of the total) was found to be associated with the isolated EEVs, while the remaining part was associated with the residual EEVs-free plasma (Fig. 2). Subsequent experiments were performed to exclude the possibility that only external naked DNA was linked to vesicles and/or that TTV particles were co-purified with EEVs during the purification step, and to demonstrate that TTV was really incorporated into the EEVs. To this purpose, to remove naked DNA linked to vesicles, in preliminary experiments it was identified the DNAse concentration (6 unit/μg of DNA) active in removing control TTV DNA purified and spiked on EEVs from TTV negative samples to be used for the treatment of EEVs TTV positive. Additionally, to demonstrate that TTV was purified associated to EEVs, it was identified Triton-100 concentration of 3%, as the optimal detergent activity to disrupt EEVs samples as demonstrated by their negative elution with exosomes purification after treatment, as reported in literature [36]. Thus, purified EEVs were treated with DNAse at a concentration known to be effective at removing absorbed EEVs TTV DNA and then tested for virus content, directly or after further washes in detergent (3% Triton-100) and subsequent 5 cycles of freezing/thawing. Conversely, plasma before EEVs purification was subjected to freezing/thawing and detergent treatments (3% Triton-100) to verify the absence of TTV in such fraction in absence of EEVs to be extracted in consequence of their destruction treatment. Figure 2 shows that the above treatments (DNAse and detergent) had no effect on the amount of TTV DNA measured in the purified EEVs while they contributed to significantly reduce the TTV yield in the vesicles obtained from plasma sample treated before their extraction likely for the decreased presence of intact vesicles to be extracted. Thus, these results indicated that TTV DNA was hard to remove from exosomes and that these vesicles could really contain amounts of the virus from infected plasma.

**Fig. 3:**
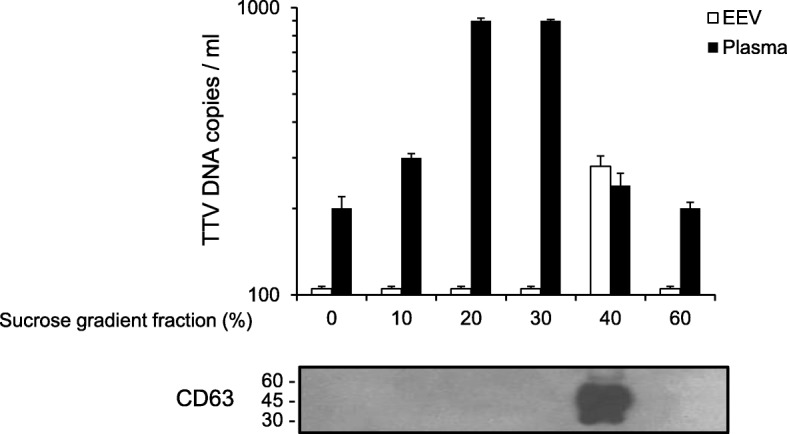
TTV load and WB of EEVs after sucrose gradient separation. TTV loads in selected plasma fraction (Plasma) and EEVs (Exosomes enriched vesicles) after sucrose gradient centrifugation are reported. The presence of CD63 positivity as EEVs representative marker in indicated sucrose gradient fraction is also indicated. The values shown are the means ± standard deviations

To furtherly confirm the association between TTV and EEVs, several experiments were performed. Firstly, total pooled plasma and purified EEVs were fractionated by using an established sucrose-density gradient ultracentrifugation procedure, and each of the fractions from 0 to 60% of sucrose was assayed for TTV presence. As shown in Fig. 3, the peak of EEVs population corresponded to the density gradient fraction of 40%, as demonstrated by the expression of exosomal marker CD63. When the content of TTV DNA was measured at the different gradient fraction, an amount of about 10^2^ DNA copies/ml was found in this peak, and this finding was consistent in all of the repeat gradients that were run.

**Fig. 4:**
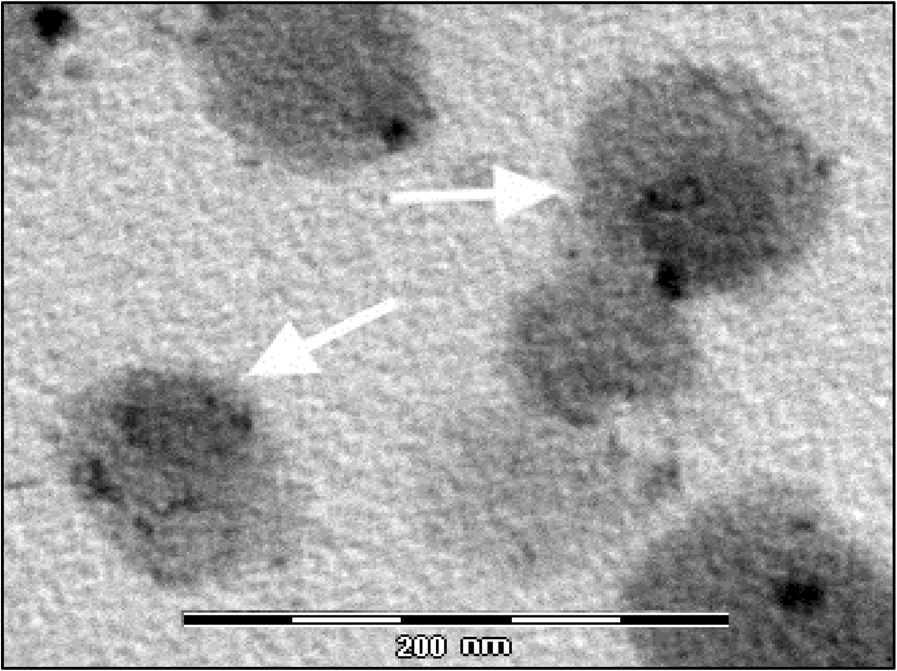
Electron microscopy of EEVs fraction. EEVs of about 90–100 nm with electron lucent core are shown. In few EEVs (white arrow), an electron dense content (~ 40–60 nm) which looks like a virus particle is observed. Calibration bar = 200 nm

Additionally, electron microscopic imaging confirmed the purity of the EEVs preparations, and evidenced the presence of proteinaceous particles (size just about of 40 nm in diameter) at least in some EEVs resembling TTV-like size (Fig. 4).

### TTV DNA presence in plasma EEVs from healthy and diseased subjects

A total of 122 subjects (97 diseased patients, and 25 healthy people) was recruited for this part of the study. Total plasma and purified EEVs samples were obtained from all these subjects and tested for TTV DNA by real-time UTR PCR. Overall, viral DNA was detected in 82 (67%) and 42 (34%) of the total plasma and EEVs samples, respectively (Table 1). The 42 virus-positive EEVs were all from viremic samples. None of the 40 plasma negative samples was found to be TTV positive in EEVs. Of the positive EEVs, 37 (88%) were from diseased patients, and the remaining from healthy people. No statistically significant differences were noted when positive EEVs were compared between healthy and diseased TTV viremic subjects (5 of 14 (36%), and 37 of 68 (54%), respectively).

**Table 1:** TTV detection in total plasma and exosomes of the study subjects, grouped by pathologies

Subjects	No. examined	No. (%) TTV DNA positive in	
		Total plasma	Exosomes
Healthy subjects	25	14 (56)	5 (20)
Diseased patients	97	68 (70)	37 (38)
HBV positive	20	14 (70)	12 (60)
HCV positive	20	12 (60)	10 (50)
HIV positive	37	30 (81)	5 (13)
Transplanted	20	12 (60)	10 (50)

When grouped by specific diseases, HIV infected patients had a positivity rate of TTV in their EEVs significantly lower than that of all other patients (13% versus 62%, respectively; Fisher exact test, *p* = 0.004), while there were no statistical differences in TTV prevalence in EEVs among HCV positive, HBV positive, and transplant patients (Table 1).

### TTV DNA load in plasma EEVs from healthy and diseased subjects

Because real-time PCR analysis showed that the levels of TTV viremia varied widely among the 80 positive individuals (mean ± error standard: 4.9 × 10^5^ ± 2.9 × 10^5^ DNA copies/ml; range: 2.0 × 10^1^–2.2 × 10^7^ DNA copies/ml), the distribution of TTV viremia detected in the above samples was compared with TTV load in EEVs. Samples positive for TTV DNA in EEVs tended to have considerably higher titres in TTV viremia than the samples negative for virus in exosomes (mean ± error standard: 9.6 × 10^5^ ± 5.6 × 10^5^ versus 1.5 × 10^3^ ± 3.2 × 10^2^ DNA copies/ml; *p* < 0.0001, Mann-Whitney test). The levels of TTV DNA found in the 42 positive EEVs were on average 4.8 × 10^3^ copies per ml (range: 1.1 × 10^1^ to 9.7 × 10^4^ copies per ml) and they were strongly correlated with the corresponding loads in total plasma (*r* = 0.700, *p* < 0.0001; Spearman Rho correlation test). Table 2 shows TTV loads in exosomes extracted vesicles from 42 subjects, grouped by different pathologies. TTV levels in vesicles from healthy people were on average lower than those in exosomes from diseased patients, but this difference, possibly for the limited number of samples tested, was not statistically significant. Similarly, TTV loads found in EEVs were similar also in all groups of diseased patients, with the highest viral loads seen in transplanted patients and the lowest in HCV positive patients. Finally, the percentage of TTV DNA harboured in EEVs of each patient was calculated by dividing the number of viral copies in exosomes with that in total plasma, multiplied by 100. As shown in Table 3, a significantly lower TTV percentage was seen in healthy controls versus diseased patients (*p* = 0.002; Mann-Whitney test), and it’s interesting to note that the percentages of TTV in EEVs varied significantly among the different groups of diseased patients (*p* < 0.001; Kruskal-Wallis test), with the highest value exhibited by transplant patients.

**Table 2:** TTV load in total plasma and exosomes in the study subjects, grouped by pathologies

Subjects	No. examined	TTV DNA copies/ml (mean ± SE)	
		Total plasma	Exosomes
Healthy subjects	5	4.4 × 10^4^ *±* 4.0 × 10^4^	2.9 × 10^2^ *±* 2.6 × 10^2^
Diseased patients	37	1.1 × 10^6^ *±* 6.4 × 10^5^	5.4 × 10^3^ *±* 3.1 × 10^3^
HBV positive	12	2.5 × 10^5^ *±* 2.1 × 10^5^	1.3 × 10^3^ *±* 8.7 × 10^2^
HCV positive	10	3.3 × 10^4^ *±* 1.2 × 10^4^	1.4 × 10^2^ *±* 3.1 × 10^1^
HIV positive	5	1.3 × 10^6^ *±* 1.1 × 10^6^	3.7 × 10^3^ *±* 3.6 × 10^3^
Transplanted	10	3.0 × 10^6^ *±* 2.2 × 10^6^	1.6 × 10^4^ *±* 1.1 × 10^4^

**Table 3:** Percentage of TTV load in exosomes in the study subjects, grouped by pathologies

Subjects	No. examined	% TTV DNA in exosomes (mean ± SE)^a^
Healthy subjects	5	1.4 *±* 0.4
Diseased patients	68	2.4 *±* 0.9
HBV positive	14	0.8 *±* 0.2
HCV positive	12	2.3 *±* 1.0
HIV positive	30	0.2 *±* 0.1
Transplanted	12	5.4 *±* 3.3

^a^Calculated from the formula: (TTV DNA copies per ml in exosomes / TTV DNA copies per ml in plasma) multiplied by100

### TTV genetic groups in EEVs

Characterization using 5 separate genogroup-specific PCR assays of the TTV present in total plasma and EEVs from 24 of the study patients is shown in Table 4. Of the EEVs specimens, 16 (67%) were infected with more than one genogroup, the remaining 8 samples harboured a single TTV genogroup. Genogroup 3 was most common (23 samples), followed by genogroups 4 (13 samples), 1 (12 samples), and 5 (5 samples). No genogroup 2 was found. Characterization of plasma TTV specific genogroup number gave identical results as those for the exosome TTV in 9 cases. The others showed different forms of discordance, although generally the number of TTV groups was less in EEVs than in the corresponding plasma samples. Interestingly, in 4 cases it was found that EEVs contained a TTV genogroup that was not seen in that plasma sample. This finding demonstrates that exosomes, at least in some cases, exhibit a TTV composition genetically different from that in plasma, thus suggesting that certain TTV groups might be associated to EEVs more frequently than other groups.

**Table 4:** TTV genogroups in plasma and exosomes samples of 24 study subjects

Category	No. (%) of TTV genogroups in	
	Plasma	Exosomes
No. of TTV genogroups		
1	3 (12)	8 (33)
2	4 (17)	6 (25)
3	11 (46)	7 (29)
4	6 (25)	3 (13)
TTV genogroup present		
Genogroup 1	20 (83)^a^	12 (50)
Genogroup 2	0 (0)	0 (0)
Genogroup 3	22 (92)	23 (96)
Genogroup 4	15 (62)	13 (54)
Genogroup 5	11 (46)	5 (21)

^a^Statistically significant from the number of genogroup 1 in exosomes (*p* = 0.014; Fisher Exact test)

### In silico analysis of the capsid ORF gene of different TTVs for detection of late assembly domains

Since different TTVs vary widely in nucleotide composition and sequence, it is really possible that they also differ in the ability to be present in exosomes vesicles. Since the potential association of virus in EVs can be related to the presence of late assembly domains PPxY and YPxL, this possibility was examined by scanning the genomes of all TTVs for published consensus sequences. This investigation, on the basis of their frequencies, could be relevant to hypothesize possible different relationships between TTV groups and plasma EEVs. Full-length or near full-length sequences present in GenBank which were representative of all the 29 TTV species currently recognized and included in the 5 genetic groups were evaluated. As shown in Table 5, both domains were found in the capsid ORF gene of TTV genome, but their prevalence varied widely among the different genogroups. PPxY domain was revealed in 9 TTV species, 7 (78%) of which included into genogroup 3, while TTV species that carried the YPxL domain were distributed among four of five genogroups. Variation in the presence of the amino acidic domains was high even within the species of a same genogroup, but interestingly 2 of 29 species, i.e. TTV 11 of group 5 and TTV 15 of group 3, showed the simultaneous expression of both domains in their capsid ORF genes.

**Table 5:** Late assembly domains in ORF1 gene of TTV genomes

TTV	No. species included	No. (%) of amino acidic motifs in ORF1 gene	
		PPxY	YPxL
Genogroup 1	5	0 (0)	1 (20)
Genogroup 2	3	0 (0)	0 (0)
Genogroup 3	12	7 (58)	3 (25)
Genogroup 4	5	1 (20)	2 (40)
Genogroup 5	4	1 (25)	3 (75)

## Discussion

The discovery of TTV as a ubiquitous virus in human body has generated concerted efforts to develop a greater understanding of the virus. To date such efforts have elucidated some aspects of the biological cycle of TTV but they have been disappointing in determining other aspects, for example what mechanisms TTV uses for spreading out so widely in organs e/o tissues of infected host. In this study preliminary experiment reported that a small percentage of TTV DNA present in plasma (6.3 × 10^2^ copies per ml, 0.65% of the total TTV DNA in plasma) was associated to well characterized EEVs. Moreover, TTV DNA was hard to remove from EEVs by DNAase treatment and was not separated from the peak of EEVs population using sucrose-gradient ultracentrifugation procedure. Conversely, destruction of EEVs contents of plasma before their extraction completely reduced the TTV DNA contents. Finally, electron microscopic imaging confirmed the presence of EEV preparation, and at least in some EEVs seemed to evidence within them the presence of small particles with size about of 40 nm in diameter, resembling to TTV virions size. However, further investigations by using more specific techniques (i. e. immunogold staining) have to be performed for confirming this evidence more clearly.

Then, the investigation was extended to plasma EEVs from 122 subjects (97 diseased and 25 healthy subjects) reporting TTV DNA detection in 34% of the samples at a mean level of 4.8 × 10^3^ copies per ml, thus confirming that plasma EEVs are able to entrap the virus. Accumulating evidence demonstrates that many viruses hijack EVs pathways to ensure their survival and persistence, and that EVs have to be considered as important mediators for virus infection-associated intercellular communication and microenvironment alteration [37–40]. In this context, EVs can mediate virus egress from cells in the absence of lysis, facilitate infection of new susceptible and/or unsusceptible cells, and favor viral escape from the immune responses. The real significance of TTV and EEVs association is not yet known, but some hypotheses can be done starting from the findings of this study. First, TTV could utilize host EEVs as a vehicle for infecting naive healthy cells, thus increasing its potential of spreading, although the low prevalence of TTV detected in EEVs, seems to reveal that this is not the major mode with which TTV spreads in the host. The use of EEVs is well known for many viruses: for example, infectious particles of non-enveloped viruses such as HAV and HEV can be engulfed by host membranes that resemble exosomes and as such are secreted from infected cells in a “quasi-enveloped” structure that permits a different way of cell entry and a wider spread in the host [41]. In this context, the acquisition of a “quasi-enveloped” structure could facilitate the entry of TTV in otherwise non-permissive cells and permit also its diffusion in immunologically privileged site such as the central nervous system [3, 42]. Second, as reported for many viruses (i.e. HEV, HAV, and picornavirus), hiding within EVs for non-enveloped viruses is a barrier to neutralizing antibodies [37, 38, 40]. Thus, EEVs might shield TTV from neutralizing antibodies, acting as a possible mechanism of immune evasion. In fact, it’s well established that TTV infected hosts mount detectable antiviral antibodies which fail to eradicate the virus, at least in the great majority of cases, and is also unsuccessful at protecting against superinfections sustained by heterologous TTV types [2, 3]. Third, since no difference was seen between healthy subjects and diseased patients, TTV and EEVs association seems to be independent from the clinical status of analyzed individuals. However interestingly, TTV loads in EEVs from immunosuppressed patients (i.e. HIV positive and transplant patients) were higher to those in healthy people and other diseased patients, thus suggesting that the amount of TTV vehiculated by EEVs could be influenced by the status of host immunity. Thus, the carriage of TTV into EVVs could be a strategy that the virus uses to reduce the level of danger signals produced by cell lysis during the TTV egress from infected cells and modulate the inflammatory response. Noteworthy, this strategy has been reported adopted by a naked virus such HEV [40]. That the active TTV replication can have effects on biological processes, which lead to inflammation in the body or in specific districts within it, is suggested by many findings. Zheng et al. [43] have demonstrated that ORF2 protein of TTV has the potential to interfere with the activity of NF-kB, a well-characterized intracellular signal transcription factor known to play a role in regulating the inflammatory response. Kincad et al. [6] have showed that the expression of a TTV-encoded miRNA can play a role in inhibiting interferon expression via the jak-stat pathway. Additionally, TTV DNA was found to provoke a dose-dependent expression and production of pro-inflammatory cytokines by robust activation of TLR-9 in ex vivo grown mouse spleen cells, thus suggesting that the resulting effect of infection on the body’s inflammatory status may vary greatly depending on the levels of TTV replicating in the host [44].

The last intriguing hypothesis suggested by the present study is that EEVs may play a role in the intra-host dissemination of the genetically different forms of TTV. To date, it has been demonstrated that miRNAs encoded by different TTVs are differently present in exosomes of viremic and not viremic subjects [29]. This could mean that the ability to release miRNAs from infected cells is different among the different forms of TTV. Thus, it cannot be excluded that certain TTV isolates can be released from infected cells by EVs easier than other isolates, having a more marked tropism for EVs. Interestingly, TTV group 3 was found to be the most prevalent in EEVs, and in some cases, the spectrum of TTVs associated to EEVs was different from the one revealed in the corresponding plasma, thus also excluding problems of contamination due to molecular protocol used. The different presence of late assembly domains in capsid ORF gene of TTV genomes could explain the observed different distribution of the TTV groups in EVs. PPXY and YPXL amino acid motifs are the main classes of late assembly domains present in viral proteins that, by interacting with host factors, are involved in the endosomal sorting complexes responsible for transport pathway in the shedding of exosome vesicles from plasma membrane for different type of virus [45]. PPxY domain was found to be more prevalent in TTV species included in genetic group 3, thus explaining the high detection of this group of TTV in EEVs and, perhaps, also its elevated prevalence in infected humans. Another intriguing finding of the study was that most EEV specimens were simultaneously infected with more than one TTV group. This demonstrates that a collective transport of genetically different TTV particles together in vesicles in circulation is a frequent event in infected subjects and suggests that the simultaneous entry of multiple TTVs genomes into a host cell using vesicular cell-uptake pathway could occur commonly. As reported for other viruses (i.e. poliovirus, coxsackievirus, and rhinovirus), the delivery of multiple virus types carried by EVs allows significantly greater replication efficiency than infections with similar numbers of viral particles not embedded in a vesicle [27]. Thus, vesicular travel of multiple viral species may be the way by which TTV generates higher levels of infection and enhances its propagation into the body of infected host.

The study presents some limitations, in particular investigations shall be extended to more subjects than in this pilot study, and repeated samples over a period of time shall be analyzed for investigating possible fluctuations of the TTV in EEVs.

## Conclusion

Collectively the results obtained provide, for the first time, a novel insight into an alternative intra-host way of TTV transmission and improve our knowledge of the mechanisms TTV has evolved for its persistence in human population.

## Abbreviations

EEVs: Exosomes enriched vesicles
EM: Electron microscopy
EV: Extracellular vesicles
HAV: Hepatitis A virus
HBV: Hepatitis B virus
HCV: Hepatitis C virus
HEV: Hepatitis E virus
HIV: Human immunodeficiency virus
miRNA: microRNA
NTA: Nanoparticles tracking analysis
ORF: Open reading frame
TTV: Torquetenoviru
UTR: Untranslated region
WB: Western blot
